# Antitumor Effect of Brusatol in Acute Lymphoblastic Leukemia Models Is Triggered by Reactive Oxygen Species Accumulation

**DOI:** 10.3390/biomedicines10092207

**Published:** 2022-09-06

**Authors:** Joana Jorge, Nisa Magalhães, Raquel Alves, Beatriz Lapa, Ana Cristina Gonçalves, Ana Bela Sarmento-Ribeiro

**Affiliations:** 1 Laboratory of Oncobiology and Hematology (LOH) and University Clinic of Hematology, Faculty of Medicine University of Coimbra (FMUC), University of Coimbra, 3000-548 Coimbra, Portugal; 2 Coimbra Institute for Clinical and Biomedical Research (iCBR)—Group of Environmental Genetics of Oncobiology (CIMAGO), Faculty of Medicine University of Coimbra (FMUC), University of Coimbra, 3000-548 Coimbra, Portugal; 3 Center for Innovative Biomedicine and Biotechnology (CIBB), 3004-504 Coimbra, Portugal; 4 Clinical Academic Center of Coimbra (CACC), 3000-061 Coimbra, Portugal; 5 Hematology Service, Centro Hospitalar e Universitário de Coimbra (CHUC), 3000-061 Coimbra, Portugal

**Keywords:** oxidative stress, NRF2 inhibitors, acute lymphoblastic leukemia, reactive oxygen species, mitochondria

## Abstract

Acute lymphoblastic leukemia (ALL) is one of the most common hematological malignancies at pediatric ages and is characterized by different chromosomal rearrangements and genetic abnormalities involved in the differentiation and proliferation of lymphoid precursor cells. Brusatol is a quassinoid plant extract extensively studied due to its antineoplastic effect through global protein synthesis and nuclear factor erythroid 2-related factor-2 (NRF2) signaling inhibition. NRF2 is the main regulator of cellular antioxidant response and reactive oxygen species (ROS), which plays an important role in oxidative stress regulation. This study aimed to evaluate the effect of brusatol in in vitro models of ALL. KOPN-8 (B-ALL), CEM (T-ALL), and MOLT-4 (T-ALL) cell lines were incubated with increasing concentrations of brusatol, and the metabolic activity was evaluated using the resazurin assay. Flow cytometry was used to evaluate cell death, cell cycle, mitochondrial membrane potential (Δψ_mit_), and to measure ROS and reduced glutathione (GSH) levels. Our results show that brusatol promoted a decrease in metabolic activity in ALL cell lines in a time-, dose-, and cell-line-dependent manner. Brusatol induced a cytostatic effect by cell cycle arrest in G_0_/G_1_ in all cell lines; however, cell death mediated by apoptosis was only observed in T-ALL cells. Brusatol leads to an oxidative stress imbalance by the increase in ROS levels, namely, superoxide anion. Redox imbalance and cellular apoptosis induced by brusatol are highly modulated by mitochondria disruption as a decrease in mitochondrial membrane potential is detected. These data suggest that brusatol might represent a new therapeutic approach for acute lymphoblastic leukemia, particularly for ALL T-cell lineage.

## 1. Introduction

Acute lymphoblastic leukemia (ALL) is a heterogeneous group of hematopoietic neoplasias that results from the malignant transformation and proliferation of lymphoid progenitor cells blocked at an early stage of differentiation in the bone marrow and that are capable to invade peripheral blood and extramedullary sites. It is mainly considered pediatric leukemia, with approximately 60% of cases diagnosed before the age of 20 years old, with a peak at 2 to 5 years old, but it can also occur in adults [1]. According to the World Health Organization (WHO, 2016), ALL may be classified as B-cell lymphoblastic leukemia/lymphoma (B-ALL) and T-cell lymphoblastic leukemia/lymphomas (T-ALL). B-ALL is further classified as B-ALL with recurrent genetic abnormalities and B-ALL is not otherwise specified [2]. ALL is highly heterogenous and harbors distinct genetic alterations, including mutations, aneuploidy, and chromosomal rearrangements that alter gene expression or encode chimeric fusion proteins [1,3]. These genes encode transcription factors, epigenetic regulators, or tyrosine kinases that disrupt several signaling pathways such as RAS-MAPK, PI3K-AKT, and JAK-STAT, as well as cell cycle regulation and epigenetic modifications, contributing to abnormal lymphoid development and differentiation [4,5]. Besides the patient’s clinical characteristics, disease-related factors, such as cytogenetic/genetic alterations, are the backbone for ALL risk assessment and stratification [1,2]. Despite the improvement of risk-adapted chemotherapeutic regimens that ameliorated the overall survival, the outcome for ALL patients with a recurrent or resistant disease remains a major concern.

Oxidative stress (OS) is involved in cancer development, namely in leukemogenesis and therapy resistance [6,7]. Nuclear factor erythroid 2-related factor-2 (NRF2) is a key transcription factor that regulates multiple pathways, for instance, glutathione synthesis, reactive oxygen species (ROS) scavenging, drug excretion and detoxification, and NADP synthesis [8]. It is the main regulator of antioxidant responses and ROS levels, tightly controlled and regulated by its natural inhibitor, the kelch-like ECH-associated protein 1 (KEAP1) [7].

**Figure 1:**
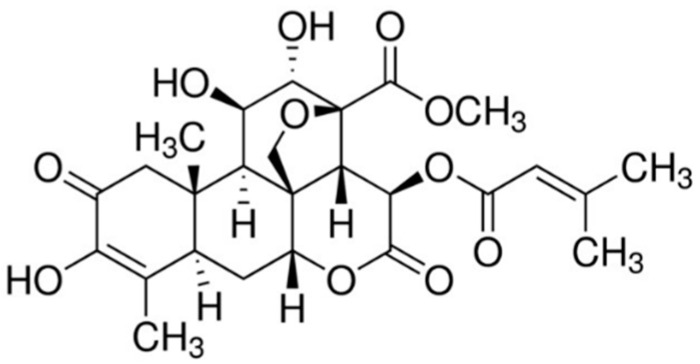
Chemical structures of brusatol.

Brusatol (Bru; Figure 1) is a quassinoid plant extract isolated from the *Brucea* species plant, capable of inducing various biological effects, including antimalarial, anti-inflammatory, and antineoplastic. Its potential as an antineoplastic drug was reported in several solid tumors, such as pancreatic cancer (PC), non-small cell lung cancer (NSCLC), renal cell cancer (RCC), among others [9,10,11,12,13,14,15,16,17], as well as in hematological malignancies and particularly acute myeloid leukemia (AML) [18]. Previous investigations demonstrated that brusatol could act as a global protein synthesis inhibitor [19], while most studies reported it as an inhibitor of NRF2 signaling by reducing its protein levels through post-transcriptional mechanisms stimulating its ubiquitination and subsequent proteolysis [20,21]. NRF2 signaling pathway inhibition by brusatol was shown to induce cell death mainly by the accumulation of ROS in NSCLC cells and PC cells [11,12]. In addition, brusatol has also been reported to sensitize different cells to chemotherapy, enhance radio-sensitivity, and overcome resistance. Ren et al. firstly demonstrated a synergistic effect of brusatol with cisplatin in in vitro and in vivo models of lung cancer [20]. This effect was later confirmed in other cancers and related to different chemo- and radiotherapeutic agents [11,18,22].

In the present study, we aimed to evaluate the antitumor effect of brusatol, an NRF2 inhibitor, in in vitro models of B- and T-cell acute lymphoblastic leukemia, including its role in oxidative stress modulation. 

## 2. Materials and Methods

### 2.1. Cell Lines Culture and Characterization

Three acute lymphoblastic leukemia cell lines were used: KOPN-8 cells, from B lineage obtained from the German Collection of Microorganisms and Cell Cultures (DSMZ, Braunschweig, Germany), and the CCRF-CEM (CEM) and MOLT-4 cell lines, from T lineage, were obtained from the American Type Culture Collection (ATTC, Manassas, VA, USA). All cell lines were maintained in Roswell Park Memorial Institute 1640 (RPMI-1640), containing 2 mM of L-glutamine, 100 U/mL of penicillin, 100 µg/mL of streptomycin (Gibco, Thermo Fisher Scientific, Waltham, MA, USA), and supplemented with 10% fetal bovine serum (FBS) (Gibco, Thermo Fisher Scientific, Waltham, MA, USA). Cells were maintained at 37 °C in a humidified atmosphere containing 5% CO_2_ and incubated at an optimal density of 0.5 × 10^6^ cells/mL (KOPN-8) and 0.3 × 10^6^ cells/mL (CEM and MOLT-4). KOPN-8 cell line was obtained from the peripheral blood of a 3-month-old girl with B cell precursor acute lymphoblastic leukemia and presented the t(11;19)(q23;p13) that leads to the *KMT2A-MLLT1* (*MLL-MLLT1*; *MLL-ENL*) fusion gene [23,24]. CEM cell line was first established from the peripheral blood of a 3-year-old girl with T-ALL at relapse and presented the *NKX2.5-BCL11B* fusion gene. The other T-ALL cell line is MOLT-4 which was established from the peripheral blood of a 19-year-old man at relapse [24]. All ALL cell lines also presented *TP53* mutations [25,26].

### 2.2. Metabolic Activity by Resazurin Assay

The resazurin assay assessed the metabolic activity of cells incubated with brusatol. This compound was purchased from Sigma-Aldrich (St. Louis, MO, USA) and dissolved in DMSO. All three cell lines were incubated for 72 h in the absence and presence of increasing concentrations of brusatol, ranging between 1 and 100 nM, using 100 times concentrated stocks to avoid DMSO cytotoxicity and to ensure consistency between conditions. The cells were plated on a 48-well plate for 72 h. Every 24 h, resazurin was added to a final concentration of 10 µg/mL and then incubated at 37 °C for 2 h. The absorbance at 570 nm and 600 nm was measured using a microplate spectrophotometer (Synergy^TM^ HT Multi-Mode Micro-plate Reader, BioTek Instruments, Winooski, VT, USA), and the metabolic activity was calculated as a percentage of control. 

### 2.3. Cell Death Evaluation

Cell death was evaluated using annexin V (AV) and 7-Aminoactinomycin D (7-AAD) double staining by flow cytometry (FC) and by morphological analysis using optical microscopy. Briefly, after 72 h of incubation in the absence and presence of brusatol, 0.5 × 10^6^ cells were washed with PBS, centrifuged at 500× *g* for 5 min, resuspended in 100 μL of annexin V binding buffer, and incubated with 2.5 μL of annexin V-APC (AV, Biolegend, San Diego, CA, USA) and 5 μL of 7-AAD (Biolegend, San Diego, CA, USA) for 15 min in the dark at room temperature (RT). Then, cells were diluted in 300 μL of annexin V binding buffer and analyzed in a FACSCalibur flow cytometer (Becton Dickinson, Franklin Lakes, NJ, USA). At least 25,000 events were acquired using CellQuest software (Becton Dickinson, Franklin Lakes, NJ, USA) and analyzed using Paint-a-Gate (Becton Dickinson, Franklin Lakes, NJ, USA). The results were expressed as a percentage of viable cells (AV^−^/7-AAD^−^), initial apoptotic (AV^+^/7-AAD^−^), late apoptotic/necrotic (AV^+^/7-AAD^+^), and necrotic cells (AV^−^/7-AAD^+^). For morphological analysis, 1 × 10^6^ untreated and treated cells were collected and seeded in glass slides. Then, smears were stained with May-Grünwald solution (Sigma-Aldrich, St. Louis, MO, USA) for 3 min, and then with Giemsa solution (Sigma-Aldrich, St. Louis, MO, USA) for 15 min. After rinsing with distilled water, cell morphology was analyzed by light microscopy using a Nikon Eclipse 80i microscope equipped with a Nikon digital camera DXm 1200F. 

### 2.4. Cell Cycle Evaluation

The cell cycle was evaluated by FC using propidium iodide (PI)/RNase cell cycle analysis kit (Immunostep, Salamanca, Spain) as previously described [27]. Briefly, after 72 h of incubation, 1 × 10^6^ of untreated and treated cells were collected and washed with PBS for 5 min at 1000× *g*. The pellet was resuspended in 200 μL of 70% ethanol solution during vortex agitation and incubated for 30 min at 4 °C. Then, cells were washed with PBS, resuspended in 500 μL of PI/RNase solution, and flow cytometry analysis was performed in a FACSCalibur flow cytometer (Becton Dickinson, Franklin Lakes, NJ, USA). Results were expressed in the percentage of cells in each cell cycle phase according to PI fluorescence intensity. A sub-G_1_ peak was also identified as apoptotic cells. The cell cycle distribution was analyzed using ModFit LT software (Verity Software House, Topsham, ME, USA). The results were expressed as a percentage of cells in the different phases of the cell cycle (G_0_/G_1_, S, and G_2_/M) and the peak sub-G_1_.

### 2.5. Oxidative Stress Evaluation

Oxidative stress levels were evaluated through the imbalance between the ROS levels and the content in antioxidant defenses, namely reduced glutathione (GSH), as described by Gonçalves et al. [28]. Intracellular peroxides and superoxide anion were measured using the dyes 2,7-dichlorodihydrofluorescein diacetate (DCFH_2_-DA; Molecular Probes, Thermo Fisher Scientific, Waltham, MA, USA) and dihydroethidium (DHE; Molecular Probes, Thermo Fisher Scientific, Waltham, MA, USA)), respectively. After 72 h, 1 × 10^6^ cells were incubated with 5 μM of DCFH2-DA for 45 min at 37 °C in a humidified atmosphere of 5% CO_2_ or with 5 μM of DHE for 15 min at RT, in the dark.

The GSH content was measured using mercury orange (MO) dye (Sigma-Aldrich, St. Louis, MO, USA) by incubating 1 × 10^6^ cells with 40 μM of MO for 15 min at RT in the dark. Cells were washed twice with cold PBS by centrifugation at 300× *g* for 5 min, resuspended in the same buffer, and analyzed by FC. Flow cytometry analysis was performed in a FACSCalibur flow cytometer (Becton Dickinson, Franklin Lakes, NJ, USA). At least 25,000 events were acquired using CellQuest software (Becton Dickinson, Franklin Lakes, NJ, USA) and analyzed using Paint-a-Gate (Becton Dickinson, Franklin Lakes, NJ, USA). The results were presented using the probe’s mean fluorescence intensity (MFI) in each condition. ROS/GSH ratio was calculated using MFI values of ROS (peroxides plus superoxide anion) and GSH.

### 2.6. Mitochondrial Membrane Potential Evaluation

The mitochondrial membrane potential (Δψ_mit_) was evaluated by FC using the fluorescent probe 5, 5′, 6, 6′ -tetrachloro-1, 1′, 3, 3′-tetraethylbenzimidazolcarbocyanine iodide (JC-1, Enzo Life Sciences, Farmingdale, NY, USA). After 72 h of incubation in the absence and presence of brusatol, 1 × 10^6^ cells were washed with PBS by centrifugation for 5 min at 300× *g* and incubated at 37 °C for 15 min with 5 µL of JC-1. Then, the cells were washed in PBS, resuspended in 300 µL, and analyzed by flow cytometry. Results were presented as JC-1 aggregate/monomer ratio that allows for comparative measurements of Δψ_mit._

### 2.7. NFE2L2 and KEAP1 Gene Expression Analysis

*NFE2L2* and *KEAP1* gene expression levels were determined as previously described [29]. Briefly, total RNA from the cell lines was extracted using NZYol reagent (NZY Tech, Lisbon, Portugal) and reversed transcribed using NZY First-Strand cDNA Synthesis Kit (NZY Tech, Lisbon, Portugal). Gene expression levels were performed by real-time quantitative PCR (qPCR) in a QuantStudio™ 5 System (Thermo Fisher Scientific, Waltham, MA, USA) using Xpert Fast SYBR 2x (GRiSP, Oporto, Portugal) with the following primers: *NFE2L2* F 5′- CAA CCC TTG TCA CCA TCT CAG-3′, *NFE2L2* R 5′- CCA GGA CTT ACA GGC AAT TCT T-3′, *KEAP1* F 5′- GCT GTC CTC AAT CGT CTC CTT-3′, *KEAP1* R 5′- GCT GTC CTC AAT CGT CTC CTT-3′, *HPRT* F 5′- CCCTGGCGTCGTGATTAGTG-3′, and *HPRT* R 5′-TCGAGCAAGACGTTCAGTCC-3′. Standard curves were created for all studied genes using a serially diluted control sample to assess the reaction efficiency. For each experiment, was a no-template control (NTC) as the negative control was included. The specificity of qPCR reactions was confirmed using the melting curve analysis. The relative expression of the target genes was analyzed using the 2^ΔCt^ formula.

### 2.8. Statistical Analysis

Statistical analysis was performed using the GraphPad Prism 9 software for Windows (GraphPad Software, San Diego, CA, USA). The IC_50_ determination was performed by non-linear curve fit dose–response. A normality test was performed with a Kolmogorov–Smirnov test, and adequate analysis was used in accordance. Student’s t-test, analysis of variance, Dunnett’s test, and Tuckey test were used to compare the different groups. A significance level of *p* < 0.05 was considered statistically significant. Results are expressed in mean ± SEM of the number of independent experiments indicated in the figure legends.

## 3. Results

### 3.1. Acute Lymphoblastic Leukemia Cell Lines Showed Different Expression Levels of NFE2L2 and KEAP1 Genes

**Figure 2:**
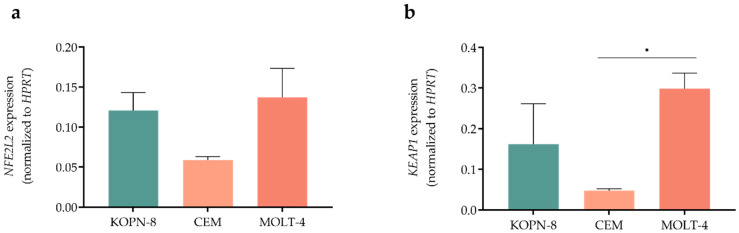
Basal *NFE2L2* (**a**) and *KEAP1* (**b**) gene expression levels in acute lymphoblastic leukemia cell lines. Results are normalized to *HPRT* gene (endogenous control) and are expressed as mean ± SEM obtained from 3 independent experiments. * *p* < 0.05.

Since NRF2/KEAP1 axis is a redox regulator and a target of brusatol, we first evaluated the expression levels of *NFE2L2* in ALL cell lines, which encodes for the NRF2 protein and *KEAP1*, the natural inhibitor of NRF2 (Figure 2). MOLT-4 cells presented a higher expression of *NFE2L2* (0.137 ± 0.036), having a two-fold higher expression than its counterpart CEM, which is the cell line with the lowest expression (0.059 ± 0.004). KOPN-8 cells presented an intermediate expression of *NFE2L2* (0.121 ± 0.022). Regarding *KEAP1* expression, the pattern of expression was similar to *NFE2L2* with MOLT-4 cells (0.298 ± 0.038), presenting a six-fold higher expression compared to CEM cells (0.047 ± 0.005, *p* = 0.042), and approximately a two-fold increase when compared to KOPN-8 cell line (0.162 ± 0.099). 

### 3.2. Brusatol Reduced Metabolic Activity in All Acute Lymphoblastic Leukemia Cell Lines

**Figure 3:**
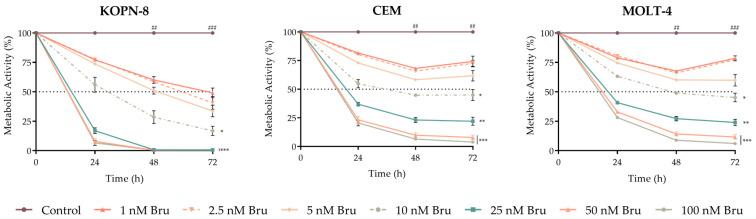
Dose–response curves of brusatol (Bru) in acute lymphoblastic leukemia cell lines. Cells were incubated with increasing concentrations of brusatol for 72 h. Data are expressed in percentage (%) normalized to control and represented as mean ± SEM obtained from at least 5 independent experiments. * *p* < 0.05, ** *p* < 0.01, *** *p* < 0.001 comparing with untreated cells (control); ## *p* < 0.01, ### *p* < 0.001 comparing with 0 h.

To assess the potential effect of brusatol on metabolic activity in ALL, the three cell lines were incubated with increasing concentrations of brusatol for 72 h, and a resazurin assay was employed at 24 h, 48 h, and 72 h. As represented in Figure 3, our results show that brusatol reduced the metabolic activity in all ALL cell lines in a dose- and time-dependent manner. However, this effect was reversed in T-ALL cells (CEM and MOLT-4), especially at lower doses.

The brusatol effect was also cell-line dependent, being the half maximal inhibitory concentration (IC_50_) of this NRF2 inhibitor, at 72 h, of 1.4 nM for KOPN-8 cells, the most sensitive cell line, and 7.4 nM for CEM and 7.8 nM for MOLT-4 cell lines (Figure 3). 

### 3.3. Proapoptotic Effect of Brusatol in Acute Lymphoblastic Leukemia Cell Lines

**Figure 4:**
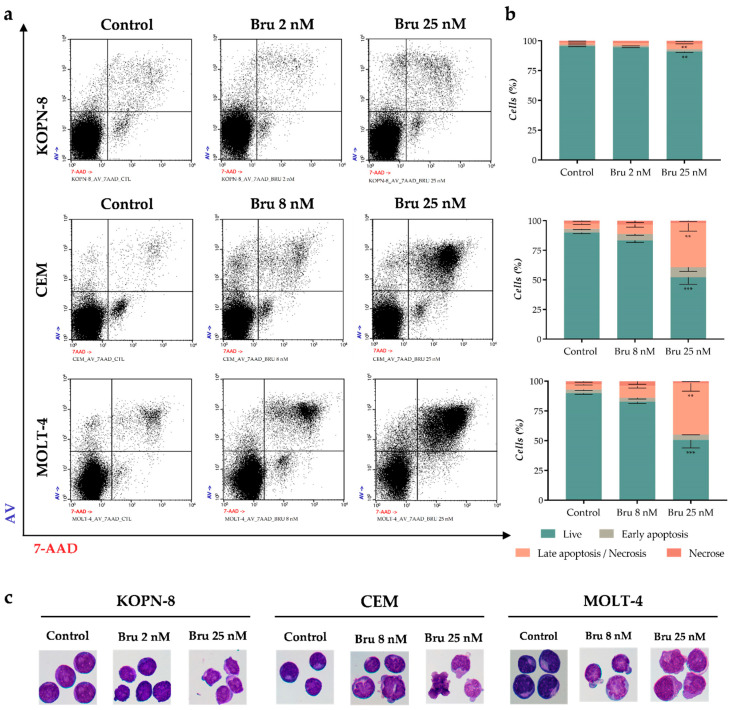
Analysis of cell death induced by brusatol in acute lymphoblastic leukemia cell lines. Cell death was detected by annexin V/ 7-AAD double staining and analyzed by flow cytometry (FC) (**a**) and (**b**) and by optical microscopy (**c**). (**a**) Representative dot plots from cell death analysis by FC; (**b**) Cell death analysis data are expressed in percentage (%) of live, early apoptotic, late apoptotic/necrotic, and necrotic cells. The data represent the mean ± SEM of at least 3 independent determinations; (**c**) Cell morphology was observed by light microscopy using May-Grünwald-Giemsa staining (amplification 500×). ** *p* < 0.01, *** *p* < 0.001 comparing with untreated cells (control). AV, annexin V; Bru, brusatol.

To further establish whether the decreased metabolic activity was induced by a cytotoxic effect of brusatol, we carried out AV/7-AAD double staining by flow cytometry, and the morphological characteristics were evaluated by optical microscopy. The cells were treated with two different concentrations of brusatol, one corresponding to the IC_50_ specific to each cell line (KOPN-8: 2 nM; CEM and MOLT-4: 8 nM), and a higher concentration of 25 nM. As represented in Figure 4a,b, the highest dose of brusatol induced a significant increase in the percentage of cells in late apoptotic/necrotic (AV^+^/7AAD^+^) in CEM and MOLT-4 cell lines (*p* < 0.01 compared to the control cells) with a concomitant decrease in the percentage of viable cells. To further establish if this effect was related to cell death by apoptosis or necrosis, the optical microscopy analysis demonstrated that cells treated with brusatol showed typical morphological features of apoptosis (Figure 4c). These features included cell shrinkage, blebbing, nuclear fragmentation, and chromatin condensation. Regarding the KOPN-8 cell line, despite the induction of a statistically significant increase in the death cell population by AV/7-AAD analysis, no biological relevance seems to be associated with brusatol treatment (late apoptotic/necrotic; control: 1.6 ± 0.3; Bru 25 nM: 4.8 ± 0.4; *p* < 0.01). However, some KOPN-8 cells treated with Bru 25 nN showed features of initial apoptosis (blebbing) in the morphological analysis (Figure 4c).

### 3.4. Cell Cycle Arrest Induced by Brusatol in Acute Lymphoblastic Leukemia Cell Lines

**Figure 5:**
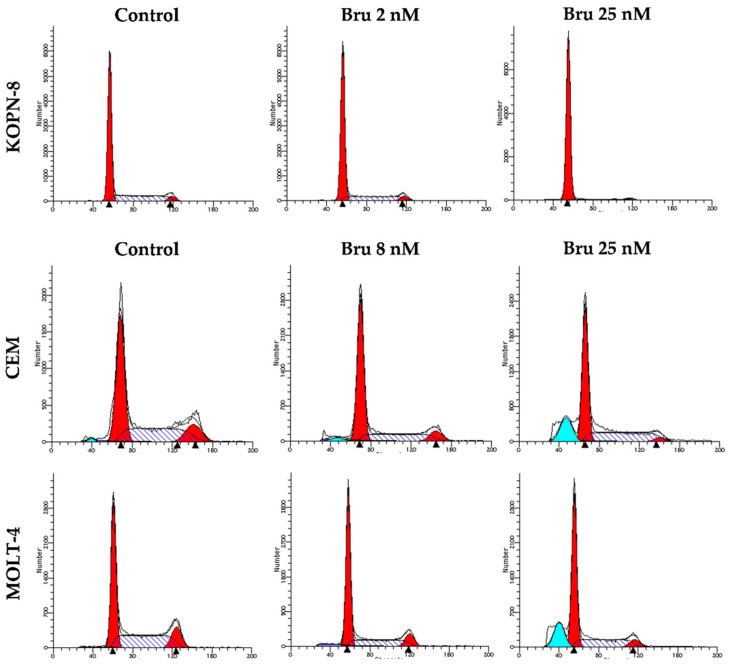
DNA-representative histograms of cell cycle analysis from acute lymphoblastic leukemia cell lines treated with brusatol. Cell cycle analysis was performed with propidium iodide/RNAse staining and analyzed by flow cytometry (FC). The initial and the last red peaks correspond to G_0_/G_1_ and G_2_/M, respectively; between them is S phase. Sub-G_1_, when present, is represented in blue peak. Bru: brusatol.

The cytostatic capacity of therapeutic compounds by the cell cycle blockage represents another critical event in cancer treatment. To assess if, besides the cytotoxic effect, brusatol also induces cell cycle arrest, cell cycle distribution was evaluated using PI/RNAse assay. As demonstrated in Table 1 and Figure 5, treatment with brusatol leads to a remarkable increase in the percentage of cells in the G_0_/G_1_ phase, in all three cell lines, and in a dose-dependent manner. 

The G_0_/G_1_ arrest was more pronounced in the KOPN-8 cell line (control: 68.4 ± 1.5; Bru 25 nM: 91.4 ± 1.3; *p* < 0.001 compared to control). Additionally, it was possible to observe the presence of a sub-G_1_ peak in CEM and MOLT-4 cells that corresponds to DNA fragmentation, another typical feature of apoptosis. This increase was statistically significant in cells incubated with 25nM brusatol (CEM: *p* < 0.001; MOLT-4: *p* < 0.05). However, and in accordance with the cytotoxic evaluation, KOPN-8 cells treated with brusatol did not present this sub-G_1_ population. This peak is observed in cells in late apoptosis. 

### 3.5. Brusatol Promotes Changes in the Intracellular Levels of ROS and GSH

As apoptosis and NRF2 inhibition are often associated with a redox imbalance, we further evaluated the intracellular levels of peroxides and superoxide anion by flow cytometry using DCFH_2_-DA and DHE fluorescent probes, respectively. The intracellular levels of GSH were also evaluated using a mercury orange probe. Brusatol altered ROS production patterns in a cell-line-dependent manner (Table 2). The peroxide levels in KOPN-8 and CEM cells were reduced without significance after treatment with brusatol, and in MOLT-4 cells no differences were observed. Superoxide anion significantly decreased in KOPN-8 cells (control: 221.6 ± 6.7; Bru 25 nM: 106.2 ± 4.3; *p* < 0.001) but increased in T-ALL cell lines, particularly in MOLT-4 cells (control: 164.6 ± 40.2; Bru 25 nM: 520.8 ± 73.6; *p* < 0.001). Nonetheless, glutathione levels decreased in all studied cell lines upon treatment with brusatol (Table 2). This GSH reduction was more significant in KOPN-8 cells (control: 113.8 ± 4.5; Bru 25 nM: 69.6 ± 2.0; *p* < 0.001).

**Figure 6:**
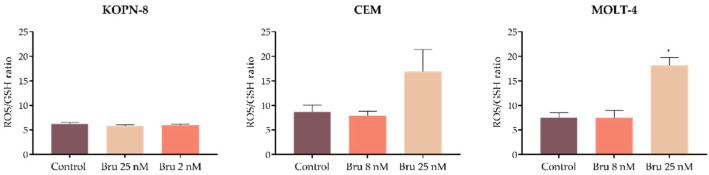
Oxidative stress evaluation by ROS/GSH ratio. Results are expressed as mean fluorescence intensity (MFI) and represent mean ± SEM obtained from at least 3 independent experiments. Bru, Brusatol; ROS; reactive oxygen species; GSH, reduced glutathione. * *p* < 0.05 compared with untreated cell (control).

To better understand the redox imbalance induced by brusatol, the cellular ROS/GSH ratio was calculated using the levels presented in Table 2. As depicted in Figure 6, in T-ALL cell lines treated with 25 nM brusatol, an increase in ROS/GSH ratio is observed, notably in MOLT-4 cells (control: 7.5 ± 1.0; Bru 25 nM: 18.1 ± 1.7; *p* < 0.05). This increase in the ROS/GSH ratio results from a redox imbalance in brusatol-treated cells favoring oxidative stress, as represented in Table 2. No differences were observed in the KOPN-8 cell line, which was in agreement with the decrease in ROS and GSH levels, described above for this cell line (Table 2). 

### 3.6. Effect of Brusatol on the Mitochondrial Membrane Potential (Δψ_mit_)

**Figure 7:**
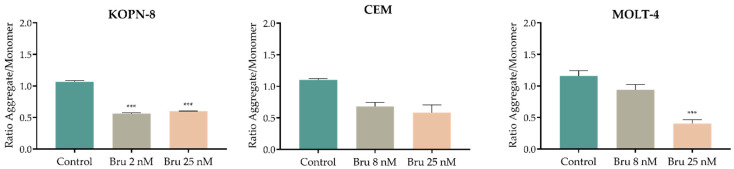
Analysis of mitochondrial membrane potential (Δψ_mit_) in acute lymphoblastic leukemia cell lines treated with brusatol. Δψ_mit_ was measured using JC-1 fluorescent probe by flow cytometry. Results are expressed in mean ± SEM of aggregate (A)/monomer (M) ratio of JC-1 and this ratio was calculated as the fraction of mean fluorescence intensity (MFI) observed for each molecule. *** *p* < 0.001 compared with untreated cell (control).

Mitochondria highly modulate the intracellular redox status and cellular apoptosis. Hence, we subsequently measured the mitochondrial membrane potential (Δψ_mit_) with JC-1 probe by flow cytometry analysis. As demonstrated in Figure 7, after brusatol treatment, a decrease in aggregates/monomers ratio (A/M ratio) was observed in all cell lines, showing a decrease in the mitochondrial membrane potential. Despite the non-significant results observed in CEM cells, both MOLT-4 (control: 1.2 ± 0.0; Bru 25 nM: 0.4 ± 0.1; *p* < 0.001) and KOPN-8 cells (control: 1.1 ± 0.0; Bru 25 nM: 0.6 ± 0.0; *p* < 0.001) presented a significant decrease in the A/M ratio and consequently in Δψ_mit_.

## 4. Discussion

Hematopoietic stem cell transplantation and a multidrug therapeutic scheme are currently the backbone of acute lymphoblastic leukemia treatment and have a considerable impact on the overall survival of these patients. Unfortunately, approximately 15% of children and a higher percentage of adults relapse after treatment or have a resistant disease [30]. For this reason, new target strategies that improve survival rates, possibly overcoming relapse and resistance, are an urgent need to ameliorate ALL treatment. Brusatol is a natural compound that has been extensively studied due to its ability to inhibit global protein synthesis and NRF2 signaling pathways, representing a good approach to cancer treatment [20]. These powerful anticancer properties were already studied in several neoplasias, such as lung cancer, pancreatic cancer, and renal cell cancer [11,12,31]. However, despite the extensive investigation on solid tumors, only a few studies focused on the impact of brusatol in leukemia. In our study, we assessed the therapeutic potential of brusatol in monotherapy in three different ALL cell lines, one derived from a B-ALL patient at diagnosis (KOPN-8) and two T-ALL cell lines at relapse (CEM and MOLT-4). We first demonstrated that brusatol has the capacity to decrease metabolic activity in all cell models. Among the three cell lines, the B-ALL was the most sensitive to brusatol, with an IC_50_ of 1.4 nM, compared to the 7.4 nM and 7.8 nM observed in both T-ALL cells, CEM and MOLT-4, respectively. Cell death was induced in CEM and MOLT-4 cells treated with higher doses of brusatol, mainly mediated by apoptosis confirmed by morphological analysis and the identification of a sub-G_1_ peak. Moreover, in our study, treatment with brusatol altered the cell cycle distribution with an accumulation of cells in the G_0_/G_1_ phase in all cell lines, with a higher impact in the KOPN-8 cell line, demonstrating the antiproliferative effect of this compound. Mata-Greenwood et al. first demonstrated that brusatol has major antiproliferative and cytotoxic effects in several leukemia cell lines, by inducing cell differentiation and G_1_ cell cycle arrest through the down-regulation of c-MYC protein expression [32]. In addition, a study by Pei et al. in a different set of leukemia cell lines demonstrated that brusatol induced cell death by suppressing the PI3K/AKT signaling pathway [33]. In acute lymphoblastic leukemia, only very recently, a study published by Wang and colleagues observed that NRF2 overexpression in B-ALL patients could decrease therapeutic response to vincristine, one of the chemotherapeutic agents commonly used in B-ALL treatment. The group further demonstrated that brusatol synergistically increased B-ALL cells sensitivity to vincristine in vitro [34]. Similarly, brusatol combinations with cytarabine, daunorubicin, and arsenic trioxide were shown to modulate drug resistance in AML [18,21]. In another study, a combination therapy of brusatol plus cytarabine also demonstrated good results in AML models specifically bearing an FLT3 internal tandem duplication [31]. The induction of cell cycle arrest in the G_0_/G_1_ phase by brusatol was already described by others in NSCLC and leukemia cell lines. Particularly, a study by Xing et al. found that brusatol might bind to 17 key proteins in cell cycle progression. This group further demonstrated that brusatol binds to SKP1, leading to the inhibition of SKP2-SCF E3 ligase, resulting in the accumulation P27, and thus G_1_ cell cycle arrest in NSCLC cells [13]. Furthermore, our study showed a redox imbalance in cells treated with brusatol with an accumulation of reactive oxygen species paired with decreased reduced glutathione, an important antioxidant element. This redox imbalance was more pronounced in T-ALL cell lines, CEM, and MOLT-4 cells. Finally, we assessed mitochondrial function and demonstrated that brusatol induced the depolarization of Δψ_mit_ in all studied cell lines, a process that is often triggered by ROS accumulation. In agreement with our findings, a study conducted on NSCLC cells revealed a possible association between brusatol and the modulation of ROS-mediated mitochondrial-dependent pathways together with the inhibition of an antioxidant response mediated by NRF2 inhibition [12]. In another study, the promotion of ROS and NRF2 suppression by brusatol resulted in a growth inhibition of lung cancer and pituitary cancer cells both in vitro and in vivo [35,36]. 

Interestingly, in our study, brusatol presented different sensitivities and exerted different cellular effects between the studied cell lines. As previously stated, KOPN-8 was the most sensitive to brusatol, mainly exerting a potent cytostatic effect. On the other hand, CEM and MOLT-4 cells presented similar responses to brusatol with overlapping IC_50_ and, in addition to a cytostatic effect, brusatol also induced mitochondrial-mediated apoptosis and redox imbalance. We first evaluated the gene expression levels of *NFE2L2* and *KEAP1* in all three cells; nonetheless, the differences observed did not explain the differences in the sensitivity to brusatol between cell lines. The main difference between these cell lines is the origin lineages; KOPN-8 is a B-cell malignancy, while CEM and MOLT-4 are both T-cell lineage leukemia. Moreover, all three cell lines are associated with different genetic abnormalities, as formerly stated. In fact, the KOPN-8 cell line presents the chromosomal translocation (11;19)(q23;p13) that encodes for *KMT2A-MLLT1* (also known as *MLL-MLLT1* or *MLL-ENL*) fusion gene [24]. *KMT2A* encodes for a histone methyltransferase involved in epigenetic regulation of blood cell development, and its deregulation is associated with leukemia initiation [3,37]. This translocation and all other rearrangements involving *KMT2A* are strongly associated with a poor treatment outcome in B-ALL young patients [37]. Recently, in a study focused on its anti-inflammatory effects, a brusatol derivative was shown to inhibit the spleen tyrosine kinase (SYK) [38], a kinase whose constitutive activation and associated signaling inhibition was already demonstrated in B-ALL with *KMT2A* rearrangements [39]. These previous results indicate that the observed effect in the KOPN-8 cell line may be related to a brusatol mechanism different than what we observed in T-ALL cell lines, for example, by inducing the inhibition of proteins dysregulated specifically in B-ALL. Additionally, due to its function as a regulator of gene expression, *KMT2A* plays an important role in cell cycle regulation [40], which possibly explains the impact that brusatol exerted in cell cycle arrest in this B-ALL cell line. To assess the possibility that *KTM2A* rearrangements are differently modulated by brusatol, other cell lines and eventually primary cultures from B-ALL patients bearing this and other B-ALL-associated genetic abnormalities should be studied. Another important aspect already described by Mata-Greenwood and its colleagues that might explain the different mechanisms triggered by brusatol observed in our study is the *TP53* status. They speculate that cells with mutant *TP53* are less sensitive to brusatol whereas wild-type *TP53* are more resistant [32]. However, in our study, all three cell lines, B- and T-cell ALL, bear different *TP53* mutations, and all are sensitive to brusatol treatment. 

Despite the different genetic and cytogenetic backgrounds of CEM and MOLT-4 cell lines, in our study, these T-ALL cells showed similar responsiveness to brusatol, as previously stated. According to the COSMIC database (cancer.sanger.ac.uk accessed on august 6, 2022), CEM cells harbors mutations in *NOTCH1*, *FLT3,* and *KRAS*, while MOLT-4 in *PTEN*, *NOTCH1*, and *NRAS*, among many others, interfering with multiple key signaling pathways critical in ALL [41]. Moreover, brusatol also interferes with some of these pathways, for instance, Wang et al. demonstrated that brusatol has an inhibitory effect on renal cancer cells by regulating PTEN-PI3K-AKT signaling [9]. Similar results were presented in another study, proving that brusatol inhibits the PI3K-AKT-mTOR pathway in hepatocellular carcinoma cells [42]. Other authors also demonstrated the importance of inhibiting MAPK-ERK and PI3K-AKT pathways due to their ability to reduce NRF2 levels and prevent therapy resistance in ALL [43]. Therefore, it is important to completely unravel brusatol mechanisms of action in these cell lines and test new drug combination schemes to improve ALL treatment approaches further. 

## 5. Conclusions

In conclusion, our data show that the natural compound brusatol has high antitumor activity in acute lymphoblastic leukemia cell lines, inducing a cytotoxic effect by apoptosis and a cytostatic effect by cell cycle arrest in the G_0_/G_1_ phase. Moreover, the imbalanced oxidative state and cellular apoptosis induced by this compound seem to be modulated by the depolarization of the mitochondrial membrane. Even though further elucidations are needed, brusatol represents a promising new target drug to be included in acute lymphoblastic leukemia treatment strategy. 

## Figures and Tables

**Table 1:** Effects of brusatol in cell cycle distribution of acute lymphoblastic leukemia cell lines.

		Sub-G_1_ (%)	G_0_/G_1_ (%)	S (%)	G_2_/M (%)
KOPN-8	Control	0.2 ± 0.2	68.4 ± 1.5	27.0 ± 1.5	4.6 ± 0.4
	Bru 2 nM	0.6 ± 0.6	71.6 ± 2.1	22.8 ± 1.7	5.8 ± 1.0
	Bru 25 nM	0.0 ± 0.0	91.4 ± 1.3 ***	6.6 ± 1.0 ***	2.0 ± 0.3
CEM	Control	1.2 ± 0.4	43.8 ± 1.6	43.6 ± 2.3	12.6 ± 0.8
	Bru 8 nM	2.6 ± 0.5	58.2 ± 2.2 *	32.4 ± 2.4	9.4 ± 1.1
	Bru 25 nM	9.2 ± 2.6 ***	62.0 ± 4.8 **	31.8 ± 4.5	6.2 ± 1.0 *
MOLT-4	Control	0.8 ± 0.3	44.4 ± 1.9	44.8 ± 2.1	10.8 ± 0.7
	Bru 8 nM	2.0 ± 0.5	59.6 ± 2.0 *	30.4 ± 0.9 *	10.0 ± 1.4
	Bru 25 nM	11.2 ± 3.4 *	60.8 ± 3.2 *	33.2 ± 3.6	6.0 ± 1.9

Data are expressed as percentage of cells in G_0_/G_1_ phase, S phase, G_2_/M phase, and apoptotic peak and represent mean ± SEM obtained from at least 3 independent experiments. * *p* < 0.05, ** *p* < 0.01, *** *p* < 0.001 comparing with untreated cell (control). Bru: Brusatol.

**Table 2:** Oxidative stress parameters induced by brusatol in acute lymphoblastic leukemia cell lines.

		Peroxides (DCFH_2_-DA Fluorescence MFI)	Superoxide Anion (DHE Fluorescence MFI)	Reduced Glutathione (MO Fluorescence MFI)
KOPN-8	Control	474.8 ± 8.2	221.6 ± 6.7	113.8 ± 4.5
	Bru 2 nM	390.2 ± 24.1	238.8 ± 11.5	106.2 ± 4.3
	Bru 25 nM	294.2 ± 10.2	106.2 ± 4.3 ***	69.6 ± 2.0 ***
CEM	Control	598.2 ± 69.0	259.2 ± 81.5	104.8 ± 14.3
	Bru 8 nM	562.0 ± 80.9	324.4 ± 65.8	115.6 ± 9.3
	Bru 25 nM	503.0 ± 69.4	466.2 ± 69.6	71.4 ± 16.3
MOLT-4	Control	585.0 ± 104.7	164.6 ± 40.2	101.2 ± 11.0
	Bru 8 nM	484.8 ± 67.3	259.2 ± 65.0	110.8 ± 13.5
	Bru 25 nM	588.4 ± 79.4	520.8 ± 73.6 ***	63.4 ± 7.1

Results are expressed as mean fluorescence intensity (MFI) and represent mean ± SEM obtained from at least 3 independent experiments. Bru, Brusatol; DCFH_2_-DA, 2,7-dichlorodihydrofluorescein diacetate; DHE, dihydroethidium; MO, mercury orange. *** *p* < 0.001 compared with untreated cell (control).

## Data Availability

All data generated or analyzed during this study were included in this published article.
